# A population-based measure of chronic disease severity for health planning and evaluation in the United States

**DOI:** 10.3934/publichealth.2020006

**Published:** 2020-02-04

**Authors:** Carol L. Stone

**Affiliations:** Institute for Families in Society, University of South Carolina, Columbia, SC, USA

**Keywords:** multiple chronic conditions, chronic disease severity, health-related quality of life, population health planning, disparities in mortality, clinical risk groups, Behavioral Risk Factor Surveillance System

## Abstract

In the healthcare sector, patients can be categorized into clinical risk groups, which are based, in part, on multiple chronic conditions. Population-based measures of clinical risk groups for population health planning, however, are not available. Using responses of working-age adults (19–64 years old) from the Behavioral Risk Factor Surveillance System for survey years 2015–2017, a population-based measure of chronic disease severity (CDS) was developed as a proxy for clinical risk groups. Four categories of CDS were developed: low, medium-low, medium-high, and high, based on self-reported diagnoses of multiple chronic conditions, weighted by hospitalization costs. Prevalence estimates of CDS were prepared, by population demographics and state characteristics, and CDS association with perceived health-related quality of life (HRQOL) was evaluated. Age-adjusted CDS varied from 72.9% (95% CI: 72.7–73.1%) for low CDS, to 21.0% (95% CI: 20.8–21.2%), 4.4% (95% CI: 4.3–4.5%) and 1.7% (95% CI: 1.6–1.8%) for medium-low, medium-high, and high CDS, respectively. The prevalence of high CDS was significantly greater (p < 0.05) among older adults, those living below the federal poverty level, and those with disabilities. The adjusted odds of fair/poor perceived HRQOL among adults with medium-low or medium-high/high CDS were 2.39 times (95% CI: 2.30–2.48) or 6.53 times (95% CI: 6.22–6.86) higher, respectively, than adults with low CDS. Elevated odds of fair/poor HRQOL with increasing CDS coincided with less prevalence of high CDS among men, minority race/ethnicities, and adults without insurance, suggesting a link between CDS and risk of mortality. Prevalence of high CDS was significantly higher (p < 0.05) in states with lower population density, lower per capita income, and in states that did not adopt the ACA. These results demonstrate the relevance of a single continuous population-based measure of chronic disease severity for health planning at the state, regional, and national levels.

## 1. Introduction

Chronic disease thinking within the public health community is shifting from individual disease-specific conditions to co-existing multiple chronic conditions (MCCs), and the relationship between those conditions [1]. The MCCs are a public health priority because they are associated with high health care costs [2], as well as social limitations [1]. The interest has led to a strategic framework for addressing MCCs, which includes a coordinated response across national programs [3]. Estimates of MCCs, however, do not adjust for the cost impact of chronic conditions on the healthcare system.

In the healthcare sector, MCCs at the individual patient level are weighted by their expected health care costs to create clinic risk group designations [4]–[7]. Software groupers can be sophisticated, considering hundreds of health conditions, as well as the number of organ systems affected and health care costs. The clinical predictors, however, are not designed for population health planning.

A potential mechanism for aligning MCCs in population health with individual clinical risk is through the Behavioral Risk Factor Surveillance System (BRFSS) [8],[9]. The BRFSS produces population-based prevalence estimates on a variety of health topics, in all states and territories across the U.S. [10]. It is used in public health for program planning and evaluation, and also has usefulness for population health planning within the healthcare sector. The BRFSS provides a wide range of state and national population-based estimates of health status, health risk and protective behaviors, as well as chronic conditions.

In addition to measures of MCCs, the BRFSS survey has been used to link MCCs to perceived health-related quality of life (HRQOL) [11],[12]. The survey has also been used to relate perceived HRQOL to other health-related topics, including insurance status [13], adverse childhood experiences [14], health equity [15],[16], and tobacco use [17]. Within the healthcare setting, similar measures of HRQOL using other methods have been correlated with MCCs [18],[19], showing that adults with multiple chronic conditions have reduced perceived HRQOL. Reduced HRQOL is a well-known predictor of mortality in the healthcare sector. For instance, studies of diabetes [20], age [21], kidney disease [22], and chronic obstructive pulmonary disease [23] have all shown an increased risk of mortality with reduced quality of life.

This study was conducted to develop and assess a novel population-based indicator of clinical risk that is based on MCCs and health care expenditures, and that uses questions from the BRFSS, which are available to all states in the U.S. The measure of chronic disease severity (CDS) is sensitive to demographics and resident geographic characteristics, and its association with perceived HRQOL and risk of mortality demonstrates the relevance of CDS for population health planning in both the healthcare and public health sectors.

## 2. Materials and methods

All outcome variables and population demographics were obtained from responses to the BRFSS of working-age adults (19–64 years old), using annual questions during survey years 2015 through 2017, combined. Assessments of the CDS were conducted using a cross-sectional observational study design. Annual survey sizes totaled 434,382 responses for year 2015; 477,665 responses for year 2016; and 444,023 responses for year 2017; with a total of 1,356,070 combined survey responses among citizen-volunteers from across the continental U.S. All responses of “Don't Know/Not Sure” or “Refused” were coded as missing. All analyses were conducted using SAS statistical software (Cary, NC). The BRFSS has been classified as EXEMPT by the Human Research Protection Office at the Centers for Disease Control and Prevention (protocol number 2988.0), and all anonymous voluntary survey responses at the state level are freely and openly available for download at https://www.cdc.gov/brfss/annual_data/annual_data.htm.

### 2.1. CDS and HRQOL

A measure of CDS for each respondent in the survey was calculated from responses to the “Chronic Health Conditions” section of the BRFSS, which is a core section of the survey offered annually [24]. The section begins with, “Has a doctor, nurse, or other health professional EVER told you that you had any of the following…?”, and then asks a series of questions about heart attack, also called a myocardial infarction; angina or coronary heart disease; skin cancer; other types of cancer; cardiovascular disease or stroke; diabetes; kidney disease (not including kidney stones, bladder infection or incontinence); chronic obstructive pulmonary disease or chronic obstructive pulmonary disease (COPD), emphysema or chronic bronchitis; asthma; a depressive disorder, including depression, major depression, dysthymia, or minor depression; and some form of arthritis, rheumatoid arthritis, gout, lupus, or fibromyalgia. Heart attack was combined with coronary heart disease, and skin cancer was combined with other cancers, totaling nine possible chronic conditions.

Each positive response to one of nine chronic conditions in the BRFSS was weighted by the national average hospitalization costs [25]–[27]. Missing or non-responses were coded as zero. A total cumulative weighted score for each respondent was then calculated, ranging from zero for no diagnoses of the queried chronic conditions, to a maximum of ten for a positive response to all of the queried chronic conditions. The resulting CDS for each respondent was a single continuous measure. For analysis, measures of CDS were grouped into the following four discrete categories, roughly based on the percent distributions of nine clinical risk groups reported by Hughes and coworkers [6]: Low severity (0–0.9000); medium-low severity (0.9001–3.575); medium-high severity (3.576–5.010); and high severity (5.011–10.000). Low severity chronic conditions included individuals who reported none of the nine chronic conditions.

Perceived HRQOL was obtained in the BRFSS from the question, “Would you say that in general your health is—excellent, very good, good, fair, or poor.” Responses were combined into two categories of fair/poor health, or good/very good/excellent (good or better) health. The question has been found to meet many of the criteria needed for quality of life indexes [12].

### 2.2. Respondent demographics

Federal poverty level was calculated from responses in the BRFSS to the following questions: “How many members of your household, including yourself, are 18 years of age or older?”; “How many children less than 18 years of age live in your household?”; “Is your annual household income from all sources – Less than $10,000? Less than $15,000? Less than $20,000? Less than $25,000? Less than $35,000? Less than $50,000? Less than $75,000? At least $75,000?” Responses to household income were categorized into five groups: Less than $15,000; $15,000–$24,999; $25,000–$34,999; $35,000–$49,999; $50,000 or more. The midpoint of each income range and total family size were used to calculate approximate household federal poverty level for year 2017 [28]. The measure was grouped into the following categories: 0–32.9%, 33.0–65.9%, 66.0–99.9%, 100.0–132.9%, 133.0–199.9%, 200.0–299.9%, and 300.0% or more.

Age in the BRFSS was obtained from the question, “What is your age?” Respondent age was available in the survey by single year and was categorized into five age groups of 19–24, 25–34, 35–44, 45–54, and 55–64 years old. Other ages were coded as missing. Characteristics of sex (male or female) and race/ethnicity (non-Hispanic white, non-Hispanic Black/African American, non-Hispanic Other, and Hispanic/Latino/a) were obtained from the following questions: “Are you...Male Female”; “Are you Hispanic, Latino/a or Spanish in origin?” and “Which one or more of the following would you say is your race?” Insurance status (insured or not insured) was obtained from the question, “Do you have any kind of health care coverage, including health insurance, prepaid plans such as Health Maintenance Organizations (HMOs), government plans such as Medicare, or Indian Health Service?” Disability (disabled or not disabled) was obtained from any one of six questions: “Are you deaf or do you have serious difficulty hearing?”; “Are you blind or do you have serious difficulty seeing, even when wearing glasses?”; “Because of a physical, mental, or emotional condition, do you have serious difficulty concentrating, remembering, or making decisions?”; “Do you have serious difficulty walking or climbing stairs?”; “Do you have difficulty dressing or bathing?”; and “Because of a physical, mental, or emotional conditions, do you have difficulty doing errands alone such as visiting a doctor's office or shopping?”

### 2.3. State characteristics

State characteristics are shown in Table 1, and were assigned to each respondent in the BRFSS based on their reported state of residence. Population density (population of adults per land square mile) was calculated for each state as the ratio of 2017 population of adult state residents [29], and land size in square miles [30]. Quartile categories of population density were created: quartile 1, 0–35.0 adults per land square mile; quartile 2, 35.1–82.1 adults per land square mile; quartile 3, 82.2–178.2 adults per land square mile; and quartile 4, 178.3–9273.2 adults per land square mile. Per capita income quartiles were generated for each state using estimates of annual per capita income [31], in 2017 inflation-adjusted dollars: quartile 1, $22,500–$27,305; quartile 2, $27,306–$29,866; quartile 3, $29,867–$33,256; and quartile 4, $34,257–$50,832. States were classified by adoption of the Affordable Care Act (ACA), as of January 1, 2015 (adopted or not adopted) [32].

States were categorized into nine geographic divisions (New England, Middle Atlantic, East North Central, West North Central, South Atlantic, East South Central, West South Central, Mountain, and Pacific) using U.S. census categories [33], as shown in Table 1.

**Table 1.** Characteristics and Assigned Quartiles of States in the Continental U.S..

State	Population Density^a^	Density Quartile	Per Capita Income^b^	Income Quartile	Adopted ACA^c^	Census Division
Alabama	74.5	2	25,746	1	Yes	East South Central
Alaska	1.0	1	50,832	4	Yes	Pacific
Arizona	47.4	2	27,277	1	No	Mountain
Arkansas	44.1	2	29,011	2	No	West South Central
California	195.4	4	26,907	1	Yes	Pacific
Colorado	41.9	2	27,305	1	Yes	Mountain
Connecticut	587.4	4	25,257	1	Yes	New England
District of Columbia	9273.2	4	35,065	4	No	South Atlantic
Delaware	387.5	4	28,450	2	No	South Atlantic
Florida	310.8	4	31,214	3	No	South Atlantic
Georgia	136.6	3	26,461	1	Yes	South Atlantic
Hawaii	174.6	3	34,712	4	Yes	Pacific
Idaho	15.4	1	39,913	4	Yes	Mountain
Illinois	178.2	3	29,600	2	Yes	East North Central
Indiana	142.0	3	34,256	3	No	East North Central
Iowa	43.2	2	36,268	4	Yes	West North Central
Kansas	26.9	1	24,774	1	Yes	West North Central
Kentucky	86.6	3	30,063	3	Yes	East South Central
Louisiana	82.1	2	28,774	2	No	West South Central
Maine	35.0	1	29,886	3	No	New England
Maryland	481.4	4	27,964	2	Yes	South Atlantic
Massachusetts	700.3	4	28,706	2	Yes	New England
Michigan	137.1	3	28,282	2	Yes	East North Central
Minnesota	53.7	2	31,476	3	Yes	West North Central
Mississippi	48.4	2	26,205	1	Yes	East South Central
Missouri	68.6	2	28,123	2	Yes	West North Central
Montana	5.6	1	32,625	3	Yes	Mountain
Nebraska	18.8	1	36,914	4	Yes	West North Central
Nevada	21.1	1	31,917	3	No	Mountain
New Hampshire	120.9	3	28,938	2	Yes	New England
New Jersey	947.1	4	28,985	2	No	Middle Atlantic
New Mexico	13.2	1	39,069	4	Yes	Mountain
New York	332.4	4	34,845	4	No	Middle Atlantic
North Carolina	163.6	3	28,761	2	Yes	South Atlantic
North Dakota	8.4	1	41,365	4	No	West North Central
Ohio	221.1	4	25,471	1	Yes	East North Central
Oklahoma	43.2	2	25,888	1	Yes	West South Central
Oregon	34.1	1	26,645	1	Yes	Pacific
Pennsylvania	226.3	4	30,410	3	Yes	Middle Atlantic
Rhode Island	815.9	4	33,128	3	No	New England
South Carolina	130.3	3	34,869	4	Yes	South Atlantic
South Dakota	8.6	1	32,511	3	No	West North Central
Tennessee	126.3	3	28,015	2	Yes	East South Central
Texas	79.9	2	24,426	1	Yes	West South Central
Utah	26.5	1	39,070	4	Yes	Mountain
Vermont	54.7	2	22,500	1	Yes	New England
Virginia	166.7	3	29,866	2	No	South Atlantic
Washington	86.5	3	32,924	3	No	Pacific
West Virginia	59.9	2	35,752	4	Yes	South Atlantic
Wisconsin	83.1	2	30,557	3	Yes	East North Central
Wyoming	4.5	1	33,315	3	Yes	Mountain

State characteristics and quartiles were assigned as described in the Materials and Methods section.

^a^ – Population Density, 2017, adults per land square mile.

^b^ – Per capita income, 2017, dollars.

^c^ – Adopted the Affordable Care Act (ACA), as of January 1, 2015.

### 2.4. Prevalence estimates

Percent prevalence estimates were generated using the SURVEYFREQ procedure, according to methods previously described [34], creating prevalence estimates (percent) with 95% confidence intervals (95% CI). Briefly, state weights were adjusted for the combination of three survey years, according to the sample size for each survey year. The stratum variable was _STSTR, and for estimates at geographies larger than the state level, _STATE was added as a stratum. Except where noted, all prevalence estimates had a coefficient of variation less than 10.0%, and the confidence interval of prevalence estimates with a coefficient of variation that was at least 10.0% was suppressed (NA). Age-adjusted prevalence estimates, by state, were created based on the 2000 U.S. population [35], and using the SURVEYREG procedure, for five age categories of 19–24, 25–34, 35–44, 45–54, and 55–64 years old. Cumulative percent prevalence estimates, by state, were prepared by summing the percent prevalence estimate of each state for high, medium-high, medium-low, and low CDS, respectively. All comparisons of prevalence estimates were evaluated at the p = 0.05 level.

### 2.5. Logistic regression analysis

Bivariate and multivariate logistic regression analyses were conducted with the SURVEYLOGISTIC procedure to analyze the crude/unadjusted and adjusted association of CDS (exposure) with perceived HRQOL (outcome), creating odds ratios (OR_crude_ or OR_adj_), with 95% confidence intervals (95% CI). Adjusted multivariate regression was conducted with the covariates disability status (not disabled *versus* disabled), federal poverty level (300% *versus* 200.0–299.9%, 100–199.9%, or less than 100%), age (19–44 years old *versus* 45–54 years old or 55–64 years old), sex (female *versus* male), insurance status (insured *versus* not insured), and race/ethnicity (non-Hispanic white *versus* non-Hispanic Black/African American or Other Race/Ethnicity). For these analyses, age, CDS, and race/ethnicity were combined into three categories each and federal poverty level was combined into four categories to create balanced estimates with no more than 5% coefficient of variation. The individual contribution of covariates to the regression model was confirmed by Wald Statistic (p < 0.05), and goodness-of-fit for the logistic regression model was evaluated by likelihood ratio. All comparisons of odds ratios were evaluated at the p = 0.05 level.

### 2.6. Age-dependent estimation of chronic disease severity (CDS)

Average CDS in the population was obtained from the BRFSS responses using the SURVEYMEANS procedure, for adults 19–24, 25–34, 35–44, 45–54, and 55–64 years old. These estimates were then fit to a generalized sigmoidal curve [36], using the NLIN procedure. The equation had the form: CDS = A + (K − A)/((1+e^−B(age − 19)^)^1/V^), where A = 0.382 (fixed), and K = 2.68 (fixed). Criteria for the fit was an iterative variation of less than 0.0001 for two estimated coefficients, which produced the following values: B = 0.0736 (95% CI: 0.0593–0.0879); and V = 0.1015 (95% CI: 0.0872–0.1158).

## 3. Results

### 3.1. Prevalence of CDS in the continental United States, by demographics

The percent prevalence in the continental U.S. for working adults (19–64 years old) of low (A), medium-low (B), medium-high (C), and high (D) levels of CDS, by population demographics, is shown in Table 2. Whereas 63.9% (95% CI: 63.8–64.1%) of adults had low CDS, the prevalence of medium-low, medium-high, and high CDS was 25.8% (95% CI: 25.7–26.0%), 7.2% (95% CI: 7.1–7.3%), and 3.0% (95% CI: 2.9–3.1%), respectively. The age-adjusted prevalence was 72.9% (95% CI: 72.7–73.1%), 21.0% (95% CI: 20.8–21.2%), 4.4% (95% CI: 4.3–4.5%), and 1.7% (95% CI: 1.6–1.8%), for low, medium-low, medium-high, and high CDS, respectively.

Prevalence of high CDS increased with age (Table 2), from a low of 0.5% (95% CI: 0.4–0.6%) among 30–39 year olds, to a high of 5.6% (95% CI: 5.3–5.8%) among adults 60–64 years old. Compared to 30-39 year olds, the prevalence of low CDS was significantly less in all older age groups (p < 0.05), and the prevalence of medium-low, medium-high, and high CDS was significantly greater (p < 0.05). Overall, the distribution of CDS shifted from a greater prevalence of low CDS among younger adults, to a greater prevalence of high CDS among older adults.

**Table 2.** Percent Prevalence of CDS in the United States, 2015–2017, combined, By Demographics, Adults 19–64 years old.

	A. Low.			B. Medium-Low			C. Medium-High			D. High		

	N	Percent	95% CI	N	Percent	95% CI	N	Percent	95% CI	N	Percent	95% CI
Total	731,320	63.9	63.8–64.1	427,843	25.8	25.7–26.0	137,868	7.2	7.1–7.3	59,039	3.0	2.9–3.1
Age-Adjusted^1^		72.9	72.7–73.1		21.0	20.8–21.2		4.4	4.3–4.5		1.7	1.6–1.8
Age												
19–29	109,279	85.6	85.3–86.0	17,730	13.2	12.9–13.5	1453	1.0	0.9–1.1	222	0.1^a^	NA
30–39	118,214	81.7	81.4–82.0	25,287	15.6	15.3–15.9	3939	2.1	2.0–2.2	926	0.5	0.4–0.6
40–44	56,339	74.6	74.1–75.2	16,299	20.0	19.5–20.5	3357	4.1	3.8–4.4	994	1.2	1.0–1.3
45–49	61,119	68.2	67.6–68.7	23,164	24.5	24.0–25.1	5414	5.3	5.0–5.6	2004	2.0	1.8–2.2
50–54	6887	60.6	60.0–61.1	34,574	28.7	28.2–29.2	9466	7.6	7.3–7.9	3983	3.1	3.0–3.3
55–59	71,739	52.6	52.1–53.2	46,530	32.9	32.4–33.4	14,471	10.0	9.7–10.3	6394	4.5	4.3–4.7
60–64	68,386	45.3	44.8–45.9	56,961	36.9	36.4–37.4	18,856	12.2	11.9–12.5	8292	5.6	5.3–5.8
Federal Poverty Level (percent)												
300 or more	267,657	62.7	62.4–63.0	164,936	28.5	28.3–28.8	43,134	6.7	6.5–6.8	13,103	2.1	2.0–2.2
200.0–299.9	157,038	68.8	68.4–69.1	76,909	23.6	23.3–23.9	22,994	5.5	5.4–5.7	9286	2.1	2.0–2.2
133.0–199.9	60,914	63.2	62.6–63.7	37,175	25.1	24.6–25.6	14,543	8.0	7.7–8.3	7255	3.7	3.5–3.9
100.0–132.9	29,990	54.9	54.1–55.6	24,078	27.7	27.0–28.4	11,937	11.1	10.7–11.5	7219	6.3	6.0–6.6
66.0–99.9	37,131	59.6	58.9–60.3	23,479	25.4	24.8–26.0	11,306	9.7	9.3–10.0	7102	5.3	5.0–5.5
33.0–65.9	18,562	63.6	62.6–64.5	9,827	23.0	22.1–23.8	4393	8.6	8.2–9.1	2619	4.8	4.5–5.2
0.0–32.9	1575	68.5	65.4–71.6	692	22.7	19.9–25.6	230	5.2^a^	NA	148	3.5^a^	NA
Race/Ethnicity^2^												
non-Hispanic white	538,812	59.4	59.2–59.5	343,836	28.7	28.5–28.8	111,766	8.5	8.4–8.6	46,164	3.5	3.4–3.6
non-Hisp Bl/African Am	58,679	63.7	63.2–64.2	33,925	26.1	25.6–26.6	11,026	7.1	6.8–7.3	5146	3.1	2.9–3.3
non-Hispanic Other	52,387	74.5	73.8–75.2	22,081	18.6	18.0–19.2	7239	4.5	4.2–4.8	4275	2.4	2.2–2.5
Hispanic/Latino/a	67,911	76.7	76.3–77.2	20,463	18.1	17.6–18.5	5369	3.7	3.5–3.9	2264	1.5	1.4–1.6
Sex^2^												
Female	385,620	59.9	59.6–60.1	258,900	28.3	28.1–28.5	87,366	8.4	8.3–8.5	36,100	3.4	3.3–3.5
Male	345,485	68.2	68.0–68.4	168,853	23.2	23.0–23.4	50,477	5.9	5.8–6.0	22,920	2.7	2.6–2.8
Insurance Status^2^												
Insured (ref)	656,911	62.0	61.8–62.2	404,368	27.0	26.9–27.2	131,709	7.7	7.6–7.8	56,537	3.3	3.2–3.4
Not insured	70,650	77.3	76.8–77.8	22,230	17.3	16.9–17.8	5856	3.9	3.7–4.1	2338	1.5	1.4–1.6
Disability Status												
Not disabled	606,845	73.7	73.6–73.9	263,022	22.0	21.9–22.2	50,468	3.5	3.4–3.6	11,173	0.8	0.7–0.9
Disabled	92,762	33.1	32.8–33.5	149,432	37.9	37.6–38.3	82,977	18.8	18.6–19.1	46,172	10.1	9.9–10.3
Perceived HRQOL												
Excellent	180,876	84.3	84.0–84.5	43,982	14.2	14.0–14.5	1371	1.2	1.2–1.4	833	0.2	0.1–0.3
Very Good	283,766	72.3	72.1–72.6	133,933	23.6	23.4–23.8	23,109	3.4	3.3–3.5	4195	0.6	0.5–0.7
Good	206,834	60.9	60.6–61.2	154,412	29.4	29.2–29.7	48,287	7.6	7.4–7.7	13,897	2.1	2.0–2.2
Fair	49,003	38.9	38.4–39.4	70,852	35.9	35.4–36.4	40,594	17.1	16.8–17.4	20,696	8.1	7.9–8.3
Poor	9365	18.5	17.8–19.2	23,284	33.4	32.6–34.1	20,656	25.8	25.1–26.4	19,139	22.3	21.7–22.9

N—Respondent sample size; Percent – percent prevalence (%); 95% CI—95% confidence interval of the percent prevalence.

Except where noted, the coefficient of variation for all estimates was less than 10%.

^a^—The coefficient of variation for the prevalence estimate was greater than 10%, and the confidence interval, therefore, was suppressed (NA).

^1^—Prevalence of chronic disease severity (CDS) was age-adjusted to the 2000 population, using five age categories: 19–24, 25–34, 35–44, 45–54, and 55–64 years old.

^2^—The greater prevalence in low CDS and less prevalence of higher CDS among males, minority race/ethnicities, and the uninsured may reflect increased mortality in these groups.

As shown in Table 2, and compared to their counterparts, the prevalence of high CDS was significantly greater among women (3.4%, 95% CI: 3.3–3.5% *versus* 2.7%, 95% CI: 2.6–2.8% for men; p < 0.05), adults living in households with lower federal poverty level (i.e, 2.1%, 95% CI: 2.0–2.2% for federal poverty level at least 300% *versus* 6.3%, 95% CI: 6.0–6.6% for federal poverty level 100–132.9%, p < 0.05), and adults living with a disability (10.1%, 95% CI: 9.9–10.3% *versus* 0.8%, 95% CI: 0.7–0.9% among adults without a disability, p < 0.05). The prevalence of high CDS was also significantly greater among adults with less than excellent perceived HRQOL (0.2%, 95% CI: 0.1–0.3% for excellent HRQOL *versus* 22.3%, 95% CI: 21.7–22.9% for poor HRQOL, p < 0.05). Individuals with these demographics had significantly less prevalence of low CDS (p < 0.05).

Adults of minority race/ethnicity had a significantly greater prevalence of low CDS and significantly less prevalence of high CDS, compared to non-Hispanic white adults (Table 2; p < 0.05). Whereas the prevalence of low CDS among non-Hispanic white adults was 59.4% (95% CI: 59.2–59.5%), the prevalence among non-Hispanic Black/African American adults was 63.7% (95% CI: 63.2–64.2%), and the prevalence among non-Hispanic Other and Hispanic/Latino/a adults was 74.5% (95% CI: 73.8–75.2%) and 76.7% (95% CI: 76.3–77.2%), respectively. Conversely, whereas the prevalence of high CDS among non-Hispanic white adults was 3.5% (95% CI: 3.4–3.7%), the prevalence among non-Hispanic Black/African American, non-Hispanic Other, and Hispanic/Latino/a adults was 3.1% (95% CI: 2.9–3.3%), 2.4% (95% CI: 2.2–2.5%), and 1.5% (95% CI: 1.4–1.6%), respectively. Compared to adults with insurance, adults without insurance had significantly greater prevalence of low CDS and less prevalence of high severity conditions (p < 0.05).

### 3.2. Association between CDS and perceived fair/poor HRQOL

To understand if high levels of CDS were associated with increased likelihood of perceived fair/poor HRQOL, the association between CDS and fair/poor HRQOL was evaluated (Table 3). The CDS measure was strongly associated with fair/poor HRQOL, and the odds of this outcome increased with increasing severity of conditions. Compared to adults with low CDS, those with medium-low CDS had more than twice the adjusted odds of fair/poor HRQOL (OR_adj_ = 2.39; 95% CI: 2.30–2.48); and those with medium-high/high CDS had more than six-fold greater adjusted odds of fair/poor HRQOL (OR_adj_ = 6.53; 95% CI: 6.22–6.86).

Disabled adults had over four times the adjusted odds of fair/poor HRQOL, compared to adults without a disability (OR_adj_ = 4.51; 95% CI: 4.36–4.67) (Table 3). Low federal poverty level was also a strong risk factor for fair/poor HRQOL; adults living below the 200% federal poverty level had more than twice the odds (OR_adj_ = 2.37; 95% CI: 2.27–2.48), and adults living below the 100% federal poverty level had more than three times the odds (OR_adj_ = 3.39; 95% CI: 3.23–3.56), compared to adults living at or above the 300% federal poverty level.

**Table 3.** Unadjusted and Adjusted Association of CDS with Fair/Poor Perceived HRQOL United States, 2015–2017, combined, Adults 19–64 years old.

Covariate	Unadjusted		Adjusted	
	OR	95% CI	OR	95% CI
CDS (ref = Low)				
Medium-Low	3.12	3.06–3.20	2.39	2.30–2.48
Medium-High/High	11.66	11.4–12.0	6.53	6.22–6.86
Disability Status (ref = not disabled)				
Disabled	8.44	8.26–8.62	4.51	4.36–4.67
Federal Poverty Level (ref = 300% and more)				
200.0–299.9%	1.24	1.21–1.28	1.30	1.24–1.35
100–199.9%	3.01	2.92–3.10	2.37	2.27–2.48
less than 100%	4.57	4.43–4.71	3.39	3.23–3.56
Age (ref = 19–44 years old)				
45–54	1.80	1.75–1.85	1.49	1.43–1.55
55–64	2.34	2.28–2.41	1.62	1.56–1.69
Sex (ref = Female)				
Male	0.88	0.87–0.90	1.15	1.11–1.19
Insurance Status (ref = insured)				
Not insured	1.46	1.42–1.51	1.44	1.37–1.51
Race/Ethnicity (ref = non-Hispanic white)				
non-Hispanic Black/African American	1.44	1.40–1.49	1.23	1.17–1.29
Other Race/Ethnicity	1.47	1.44–1.51	1.74	1.67–1.81
Likelihood Ratio			8050.62	
degrees of freedom			18	
p value			<0.0001	

OR—odds ratio; 95% CI—confidence interval for the odds ratio (95%)

All covariates contributed significantly to the regression model (p < 0.0001).

Increasing age was associated with increasing unadjusted odds of fair/poor HRQOL (Table 3). Compared to adults 19–44 years old, adults 45–54 years old had elevated odds of fair/poor HRQOL (OR_crude_ = 1.80; 95% CI: 1.75–1.85), and adults 55–64 years old had more than twice the odds (OR_crude_ = 2.34; 95% CI: 2.28–2.41). When adjusted for CDS and other covariates, age was attenuated as a risk factor, with adjusted odds ratios of 1.49 (95% CI: 1.43–1.55), and 1.62 (95% CI: 1.56–1.69), for adults 45–54 years old, and 55–64 years old, respectively.

Adults of minority race/ethnicity and adults without insurance had increased adjusted odds of fair/poor perceived HRQOL (Table 3). The odds of fair/poor HRQOL among non-Hispanic Black/African adults were 1.23 times higher (95% CI: 1.17–1.29), compared to non-Hispanic white adults; and the odds among adults of Other race/ethnicities were 1.74 times higher (95% CI: 1.67–1.81). The adjusted odds of fair/poor HRQOL among adults without insurance were 1.44 times higher (95% CI: 1.37–1.51) than adults with insurance. Sex was a factor in HRQOL; men had greater odds of fair/poor HRQOL than women (OR_adj_ = 1.15; 95% CI: 1.11–1.19).

### 3.3. Cumulative age-adjusted prevalence of CDS, by state

**Figure 1.**
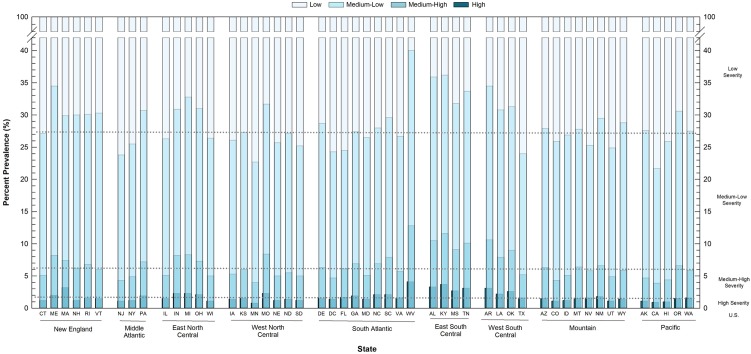
Prevalence of CDS, 2015–2017, combined, By State and Census Division, Adults 19–64 years old. Cumulative age-adjusted percent prevalence for high, medium-high, medium-low, and low severity chronic conditions are shown for all states in the continental United States. States are organized by census division. Age-adjusted prevalence estimates of each CDS level at the U.S. level are shown with dotted lines.

State-specific cumulative age-adjusted prevalence of CDS among adults of working age (19–64 years old) in the continental U.S. are shown in Figure 1. States with the greatest prevalence of high CDS included West Virginia (4.1%), Kentucky (3.7%), and Massachusetts (3.4%), followed by Alabama (3.3%), Arkansas (3.1%) and Tennessee (3.1%). Three of these states are located in the East South Central Division of the U.S., and all states in this division had prevalence estimates greater than the national age-adjusted prevalence of 1.7%, as shown earlier in Table 2.

When high and medium-high CDS prevalence were combined, all four states in the East South Central Division of the U.S., including Alabama, Kentucky, Mississippi, and Tennessee, exceeded the national cumulative prevalence of 6.1% (Figure 1). Thirteen of the 17 states that comprise the Southern Region of the continental U.S. (South-Atlantic, East South Central, and West South Central Divisions), had prevalence values at or above this national percent. Similarly, four of the six states comprising the New England Division, and three of the five states comprising the East North Central Division had cumulative prevalence values at or above the national estimate.

Cumulative high, medium-high, and medium-low CDS prevalence values further accentuated state difference (Figure 1). The state with the highest cumulative prevalence was West Virginia, in which 40.9% of the population of working age adults had at least medium-low CDS. Other states with a high percent prevalence included Kentucky (36.1%), Alabama (35.9%), Arkansas (34.5%), Maine (34.5%), and Tennessee (33.6%). In these states, at least one-third of the population of working-age adults reported at least medium-low CDS. Consequently, these areas of the country had the least prevalence of low CDS.

Twenty-one states fell below the national cumulative percent for high, medium-high, and medium-low CDS levels (27.1%; Figure 1). States with the lowest cumulative prevalence included California (21.6%), Minnesota (22.7%), and New Jersey (23.8%), as well as Texas (24.0%), District of Columbia (24.3%), Florida (24.5%), Utah (24.9%), New York (25.0%), South Dakota (25.1%), and Nebraska (25.2%). Consequently, these states had the greatest prevalence of low CDS levels.

### 3.4. Prevalence of CDS in the United States, by state characteristics

Compared to states in the continental U.S. with a high population density (178.3–9273.2 adults per land square miles), states with lower population density had significantly less prevalence of low CDS (Table 4; p < 0.05). The prevalence of low CDS among states with a high population density was 65.3% (95% CI: 65.0–65.6%), and the prevalence of medium-low, medium-high, and high CDS was 25.3% (95% CI: 25.1–25.6%), 6.7% (95% CI: 6.6–6.8%), and 2.6% (95% CI: 2.5–2.7%), respectively. In contrast, states with a low population density (less than 35.0 adults per land square miles) had significantly less prevalence of low CDS (63.9%; 95% CI: 63.6–64.2%; p < 0.05), and significantly greater prevalence of medium-low (26.2%; 95% CI: 26.0–26.5%, p < 0.05) and medium-high (7.1%; 95% CI: 7.0–7.2%, p < 0.05) severity conditions.

Compared to states of high annual per capita income, states with a medium-low or low annual per capita income had significantly less prevalence of low CDS and significantly greater prevalence of medium-high or high CDS (Table 4; p < 0.05). States with high per capita income (at least $34,257) had a prevalence for low, medium-low, medium-high, and high CDS of 65.4% (95% CI: 65.1–65.6%), 25.8% (95% CI: 25.6–26.1%), 6.3% (95% CI: 6.2–6.4%), and 2.4% (95% CI: 2.3–2.5%), respectively. In contrast, states with a low annual per capita income (less than $27,305) had a prevalence of 58.9% (95% CI: 58.6–59.2%), 27.5% (95% CI: 27.2–27.7%), 9.2% (95% CI: 9.1–9.4%), and 4.4% (95% CI: 4.3–4.5%) for low, medium-low, medium-high, and high CDS, respectively. The increasing trend in CDS was more pronounced with decreasing state-level per capita income than with decreasing population density.

Compared to states that adopted the ACA by January 1, 2015, states that did not adopt the ACA had significantly greater prevalence of medium-high and high CDS (Table 4; p < 0.05). The prevalence of low, medium-low, medium-high, and high CDS for states that adopted the ACA was 65.0% (95% CI: 64.8–65.2%), 25.7% (95% CI: 25.5–25.9%), 6.7% (95% CI: 6.6–6.8%), and 2.6% (95% CI: 2.5–2.7%), respectively. In contrast, the prevalence of these CDS levels for states that did not adopt the ACA was 63.2% (95% CI: 62.9–65.2%), 25.5% (95% CI: 25.3–25.8%), 7.7% (95% CI: 7.6–7.9%), and 3.5% (95% CI: 3.4–3.6%), respectively.

Geographic variation in CDS across the continental U.S. was also observed (Table 4). The Pacific Division of the U.S. had a significantly greater prevalence of low CDS and significantly less prevalence of medium-high and high CDS levels than all other census divisions of the continental U.S (p < 0.05). The prevalence of low, medium-low, medium-high, and high CDS among residents in the Pacific Division was 68.5% (95% CI: 68.0–69.0%), 23.6% (95% CI: 23.2–24.1%), 5.8% (95% CI: 5.6–6.0%), and 2.1% (95% CI: 2.0–2.2%), respectively. In the East South Central division of the country, these prevalence estimates were 56.4% (95% CI: 55.9–56.9%), 28.2% (95% CI: 27.8–28.7%), 10.2% (95% CI: 9.9–10.5%), and 5.2% (95% CI: 5.0–5.4%), respectively.

**Table 4.** Prevalence of CDS, United States, 2015–2017, combined, By State Characteristics, Adults 19–64 years old.

	CDS Percent Prevalence											
	A. Low			B. Medium-Low			C. Medium-High			D. High		
			
	N	Percent	95% CI	N	Percent	95% CI	N	Percent	95% CI	N	Percent	95% CI
Population Density												
High	213,575	65.3	65.0–65.6	122,864	25.3	25.1–25.6	38,668	6.7	6.6–6.8	16,412	2.6	2.5–2.7
Medium-High	169,890	62.0	61.8–62.3	103,097	26.6	26.4–26.9	34,522	7.8	7.7–8.0	15,418	3.5	3.4–3.6
Medium-Low	175,876	63.7	63.4–64.1	105,611	25.6	25.2–25.9	35,268	7.4	7.2–7.6	15,773	3.3	3.2–3.4
Low	171,979	63.9	63.6–64.2	96,271	26.2	26.0–26.5	29,410	7.1	7.0–7.2	11,436	2.7	2.6–2.8
Per Capita Income												
High	219,652	65.4	65.1–65.6	123,728	25.8	25.6–26.1	35,797	6.3	6.2–6.4	13,860	2.4	2.3–2.5
Medium-High	142,964	65.9	65.5–66.3	79,880	25.2	24.9–25.6	23,675	6.5	6.3–6.6	9185	2.4	2.3–2.5
Medium-Low	225,504	63.7	63.4–64.0	131,423	25.5	25.3–25.8	44,293	7.5	7.3–7.6	19,677	3.3	3.2–3.4
Low	143,200	58.9	58.6–59.2	92,812	27.5	27.2–27.7	34,103	9.2	9.1–9.4	16,317	4.4	4.3–4.5
ACA Status												
Adopted	382,811	65.0	64.8–65.2	221,607	25.7	25.5–25.9	67,738	6.7	6.6–6.8	27,584	2.6	2.5–2.7
Did not adopt	269,859	63.2	62.9–63.5	162,159	25.5	25.3–25.8	56,135	7.7	7.6–7.9	25,722	3.5	3.4–3.6
Census Division												
Pacific	74,338	68.5	68.0–69.0	37,085	23.6	23.2–24.1	10,449	5.8	5.6–6.0	4030	2.1	2.0–2.2
Mountain	102,570	65.2	64.9–65.6	57,422	25.6	25.2–25.9	17,424	6.7	6.5–6.8	6591	2.5	2.4–2.6
West North Central	134,285	63.9	63.5–64.2	72,267	26.0	25.7–26.3	21,817	7.2	7.0–7.4	8602	2.9	2.8–3.0
West South Central	45,868	65.7	65.0–66.3	28,490	24.2	23.6–24.8	10,381	6.9	6.6–7.2	5085	3.2	3.0–3.4
East North Central	67,707	61.2	60.8–61.6	43,207	27.6	27.2–27.9	14,719	7.8	7.6–8.0	6651	3.4	3.3–3.5
East South Central	39,497	56.4	55.9–56.9	27,497	28.2	27.8–28.7	10,998	10.2	9.9–10.5	5691	5.2	5.0–5.4
New England	75,034	62.0	61.6–62.5	47,798	28.0	27.6–28.4	14,550	7.2	7.0–7.4	5574	2.7	2.6–2.9
Middle Atlantic	60,213	63.8	63.4–64.3	34,342	26.6	26.2–27.0	10,017	6.9	6.6–7.1	4152	2.7	2.6–2.8
South Atlantic	131,758	63.4	63.0–63.7	79,735	25.6	25.3–25.9	27,513	7.6	7.5–7.8	12,663	3.4	3.2–3.5

N—respondent sample size; Percent—percent prevalence estimate (%); 95% CI: 95% confidence interval of the prevalence estimate.

The coefficient of variation for all estimates was less than 5%.

States were classified into population density and income quartiles as described in the Methods section, and shown in Table 1.

### 3.5. Age-dependent CDS in the continental United States, 19–64 years old

**Figure 2.**
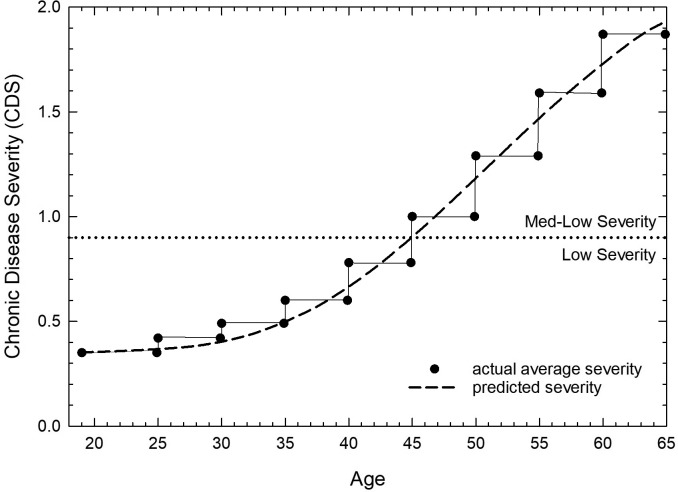
Average CDS by age. Average CDS in the continental U.S. among adults 19–64 years old is shown across discrete age groups (symbols). Predicted average CDS, by single age, was fit to these estimates, using a generalized sigmoid curve: CDS = 0.382 + (2.3)/((1 + e^(age − 19)^)^1/13.2^). See Methods section for more details. The cut-off criteria for low severity and medium-low CDS (0.900) is shown with a dotted line.

The CDS measure was created as a continuous measure, and its age-dependence was fit to a generalized sigmoid curve among adults of working age (19–64 years old) (Figure 2). The fit shows that low CDS is predicted from ages 19–30 years, followed by a slight increasing trend in CDS among adults 30–40 years old. From ages 40–60 years, a near-linear increase in CDS with age is predicted, and from 60–64 year olds, the rate of increase in CDS level begins to reach a plateau. Overall, adults 19–45 years old are predicted to have low CDS, shifting to medium-low CDS at older ages among working-age adults.

## 4. Discussion

### 4.1. Predicting population-based CDS and perceived HRQOL with the BRFSS

The CDS measure developed in this study generally behaved as expected, with increased prevalence of higher CDS among disabled adults, older adults, and adults living at poverty levels below the 200% federal poverty level (Table 2). The measure was also strongly correlated with perceived HRQOL (Table 3). Perceived HRQOL is a commonly used measure in both the healthcare [18],[19] and public health [11]–[17] sectors, and is a strong predictor of future health care expenditures [2], as well as mortality risk [20]–[23]. The strong association between CDS and perceived HRQOL reported in this study (Table 3) suggests that CDS may also be a predictor of these adverse health outcomes.

An increased risk of mortality with increasing CDS may explain the reduced prevalence of high CDS among minority race/ethnicities, males, and those living without insurance (Table 2). Lower prevalence of high CDS levels within these subpopulations may represent a disparity in mortality risk, with a public health and healthcare goal to more closely align the prevalence of these minority groups with their counterparts. These results are consistent with a reduced trend toward higher CDS predicted among working-age adults 60–64 years old (Figure 2).

The CDS measure was derived from living adults and may be a function of both age and mortality risk, in which older adults with high CDS are at greater risk of mortality. Risk for mortality is believed by some to have greater value than actual mortality, because mortality is an under-estimation of the health care burden related to chronic conditions [37]. Use of the BRFSS to create a population-based proxy for clinical risk group provides an opportunity to conduct population-based planning on the health care needs of residents living in the U.S.. More work on age-dependent CDS curves is needed across the entire lifespan of adults, and also among varying population demographics, to better understand the relationship of CDS with age and risk of mortality.

### 4.2. Geographic and population characteristics

The BRFSS survey is conducted in all states and U.S. territories, and is a readily available source of population-based health information, making possible sub-national comparisons of CDS and health care expenditures (Figure 1 and Table 4). This study suggests that states with a greater prevalence of high, medium-high, or medium-low CDS would be expected to have higher health care expenditures. This would include states with low population density and those with low per capita income. As a group, the East South Central and West South Central Divisions of the nation would be expected to have among the greatest health care burden in the U.S., due to higher levels of CDS.

The results of this study showed that high CDS prevalence was greater among states that did not adopt the ACA by January 1, 2015 (Table 4). Recent studies of ACA adoption showed a positive effect of HRQOL and health care coverage [38]–[40]. However, other studies showed that, in the short time since ACA implementation, there was little effect on access to health care [41], disparities in preventive services [42], and behavioral risk factors [43],[44]. None of these studies corrected for the degree of high CDS that was prevalent in some states, particularly the southern states of the nation that had not implemented the ACA. Possible adoption of the ACA among states with pre-existing prevalence of high CDS levels may have placed an unmanageable burden on their healthcare systems. More work over time is needed to correlate ACA adoption with changes in CDS and HRQOL.

Income was another predictor of high CDS levels. State-level characteristics of income inequality were recently studied with the Gini coefficient [45]. Results of CDS with state-level per capita income agreed with these results, indicating that states with medium-low or low per capita income had a greater prevalence of a high CDS levels (Table 4). Further, the results with respondent household federal poverty level, which considered family size, also showed greater prevalence of high CDS among adults living below 200% of the federal poverty level (Table 2). These data suggest that families with lower incomes have a greater prevalence of higher CDS levels, and a greater need for health care services.

Adults living with disabilities had a greater prevalence of higher CDS compared to adults without disabilities (Table 2). These results are consistent with previous studies showing a significant association between chronic conditions and disabilities [1]. In addition, results in this study (Table 3) compare well with other studies showing that HRQOL is reduced among individuals with disabilities [46]. Results of this study also agree with other studies of regional variation (Table 4), which showed that disabilities were geographically variant across the U.S., with a greater concentration of higher CDS levels in the Southern region of the country [47].

### 4.3. CDS and MCCs

Results of this study with CDS are comparable to those obtained with MCCs [1],[9],[18]. The advantage of CDS, however, is that, in addition to considering co-existence of MCCs, the conditions are weighted with hospitalization costs. The measure is a simplified population-based proxy for clinical risk groups that, with the BRFSS, can predict CDS in various populations. Its use, then, could be extended beyond public health to the healthcare sector. It could provide a mechanism for healthcare population planning, including Medicaid program planning [48].

The population-based discrete categories of low, medium-low, medium-high, and high CDS were developed to roughly approximate the nine clinical risk group levels developed by Hughes and coworkers [6], in which: categories of healthy, acute conditions, single minor chronic, multiple minor chronic, and single dominant or moderate chronic risk groups were comparable in prevalence to low CDS, with a percent prevalence of 74% for the clinical risk groups *versus* 72.9% (95% CI: 72.7–73.1%) for CDS (Table 2); the clinical risk category of multiple significant chronic conditions was comparable to medium-low CDS, with a percent prevalence of 21% for the clinical risk group *versus* 21.0% (95% CI: 20.8–21.2%) for medium-low CDS (Table 2); clinical risk categories of three or more dominant chronic or dominant/metastatic malignancies were comparable to medium-high CDS, with a percent prevalence of 4% for clinical risk *versus* 4.4% (95% CI: 4.3–4.5%) for medium-high CDS (Table 2); and the final risk category of catastrophic was comparable to high CDS, with a percent prevalence of 0.6% for the clinical risk group *versus* 1.7% (95% CI: 1.6–1.8%) for high CDS (Table 2). As with the clinical risk group levels, the increasing levels of CDS developed in this study are associated with increasing levels of health care expenditures.

### 4.4. Limitations

The BRFSS is a survey that relies on self-reported information from citizen-volunteers. Estimates with the BRFSS, therefore, are susceptible to self-selection bias, non-response bias, and recall bias. Reports of diagnosed chronic conditions are likely to be under-reported because they are based on respondents who have visited a health care provider. Also, the CDS was limited to the chronic conditions queried in the annual “Chronic Health Conditions” section of the BRFSS; although these conditions are considered of high health care cost and of high public health importance [49], they do not constitute all chronic conditions that may contribute to either CDS or health care costs. Some of these conditions, such as sickle cell anemia or Human immunodeficiency virus infection and acquired immune deficiency syndrome (HIV/AIDS), were not included in the measure. The CDS level for each respondent may also be under-estimated, because a missing response for any one of the queried chronic conditions was classified as a negative response and was assigned a zero value in the CDS calculation.

Estimates of CDS were weighted by average national hospitalization costs and did not take into account sub-national variations in costs for each chronic conditions. This healthcare perspective was limited to health care expenditures; use of other costs from this institutional perspective, as well as societal costs to weight the CDS calculations, could be examined. Measures of federal poverty level were based on discrete categories of income, which may have introduced bias into the results. Although this study did not explore geographies below the state level, sub-state estimates could be evaluated to assess local areas of need for greater levels of health care services, including case management services.

## 5. Conclusion

In this study, a single continuous population-based indicator of clinical risk was developed based on MCCs and health care expenditures, using the BRFSS, and making possible a readily available and freely accessible measure of CDS in populations at the state, regional, and national levels. The CDS measure among adults of working age (19–64 years old) in the U.S. showed a strong and significant independent association with perceived HRQOL, and may be a predictor of future health care expenditures and mortality risk. Its age-dependence could also be used to predict CDS across the lifespan. The CDS has utility for population health planning, and is an improved measure for assessing the ACA and other population-based health care initiatives.
